# Magnetic properties evolution and crystallization behaviour of vacuum- and air-long-term-annealed rapidly quenched Fe_80.3_Co_5_Cu_0.7_B_14_ alloy

**DOI:** 10.1038/s41598-022-25925-5

**Published:** 2022-12-10

**Authors:** L. Hawelek, T. Warski, P. Zackiewicz, J. Hudecki, A. Kolano-Burian

**Affiliations:** 1 Lukasiewicz Research Network—Institute of Non-Ferrous Metals, 5 Sowinskiego St., 44-100 Gliwice, Poland; 2 Faculty of Mechanical Engineering, PhD School, Silesian University of Technology, Akademicka 2a St, 44-100 Gliwice, Poland; 3 Laryngology Department, School of Medicine in Katowice, Medical University of Silesia, Katowice, 20-24 Francuska, 40-027 Katowice, Poland

**Keywords:** Magnetic properties and materials, Metals and alloys, Glasses

## Abstract

This work aims to investigate the isothermal crystallization behaviour, crystal structure and magnetic properties evolution of long-term (up to 300 h) low temperature (210 and 260 °C) vacuum- and air-annealed Fe_80.3_Co_5_Cu_0.7_B_14_ alloy. Before the α-Fe(Co) phase crystallization, the primary relaxation process has been identified at a temperature range up to 340 °C. The relaxation process performed under 210 °C for 300 h did not initiate the crystallization process. However, the topological and compositional short-range rearrangements improved magnetic properties remarkably. Annealing 150 h at 260 °C helps to deliver enough energy to stabilize the glassy state and initiate the crystallization process fully. Structural and magnetic properties evolution of 150 h annealing at 260 °C corresponds to the evolution presented during isochronal 20 min annealing at 310 °C. Magnetic properties Bs = 1.75–1.79 T, Hc < 20 A/m and P_10/50_ are similar to those for 20 min of annealing at 310 °C. Comparison of core power losses from up to 400 kHz frequency dependences of long-term low temperature annealed alloy with 20 min classical annealing at 310 °C shown that presented here long-term annealing is energetically insufficient to bring the glassy state system into the same low level of core power losses efficiency.

## Introduction

There are many possibilities of improving soft magnetic properties of amorphous and nanocrystalline Fe-based alloys^1–3^. Their magnetic properties are tuned by variation of the chemical composition and diverse methods of annealing process of previously obtained metallic glasses^4^. One of the very interesting Fe-based alloys groups is Co-substituted high saturation induction (Bs) nanocrystalline alloy systems^5^. For all of these alloys, two aspects are mainly taken into account: total magnetic moment per unit volume (high Fe content and Co addition to increasing the magnetic exchange interaction) and thermal stability linked with proper nanocrystallization evolution (proper Cu content)^6–9^. From the application point of view, high Bs alloys are influenced by properties of a low amorphous forming ability and relatively poor high-frequency magnetic softness (at 100 kHz and higher) that is highly desired. Lastly, many improvements in magnetic performances have been achieved by the rapid annealing process (with the heating rate of up to 1000 K/s, while standard annealing is conducted with the heating rate of 10 K/min with subsequent isothermal annealing in tenths of minutes). It has been found as an effective strategy for reducing coercivity (Hc) and core power losses (Ps) in the high-Bs alloys^10–12^. However, some Fe-based alloys have achieved optimal magnetic performance at the early stage of the crystallization process^13^, where initial α-Fe nanoclusters start to form. From this point of view, it may be concluded that some Fe-based soft magnetic materials have the best magnetic performance in the so-called relaxed state with short-ordered clusters. The question of when the glass relaxation is interrupted by crystallization and how magnetic properties evaluate is to be discussed.

According to T. Egami classification of metallic glass relaxation phenomena and description of the short-range ordering, there are two main relaxation groups. The first group shows irreversible relaxation behaviour that comprises changes in the volume, diffusivity and/or viscosity. Egami pointed out that this group of phenomena causes changes in topological short-range order. Saturation of the change characterizes the second group of relaxation phenomena after prolonged annealing. This saturated state is called the pseudo-equilibrium state since it is only metastable against crystallization^14^. T. Egami also showed it for Fe_27_Ni_53_P_14_B_6_ alloy for which the presence of compositional short-range ordering during relaxation and short-range diffusion starts to take place above 100 °C and then relaxes into equilibrium, very slowly at low temperatures, and more rapidly at higher temperatures. The reversibility of the Curie temperature (Tc) change in the temperature range of 250–300 °C has been shown during cyclic alloy annealing^15^. The changes in magnetic properties are closely related to the second group of relaxation phenomena.

The typical optimization procedure of the conventional vacuum-annealing process of Co containing Fe-Cu-B system was previously reported in^16^. In the present work, the long-term (up to 300 h) low temperature (210 and 260 °C) vacuum- and the air-annealing process is verified to determine the effects of the relaxation process on magnetic properties of high-Bs alloy Fe_80.3_Co_5_Cu_0.7_B_14._ The obtained results are also compared with optimally annealed alloy at 310 °C for 20 min. The annealing temperature was more than 30 °C below the α-Fe phase crystallization temperature onset (Tx1).

The dynamic mechanical analysis (DMA) and isothermal kinetics study have firstly been performed. Then the step-by-step long-term annealing process on toroidal cores wound from Fe_80.3_Co_5_Cu_0.7_B_14_ amorphous ribbons was optimized to find the evolution of magnetic parameters like magnetic saturation, coercivity, core power losses, maximum permeability and additionally the core power losses for frequencies up to 400 kHz. This long-term annealing approach is not currently in the leading group of soft magnetic materials researchers’ interest however, it appears to be an interesting processing method.

## Materials and methods

The amorphous alloy with nominal composition Fe_80.3_Co_5_Cu_0.7_B_14_ (at.%) in the form of ribbon with a thickness of approximately 21 µm and width of 5.6 mm were obtained via melt spinning technique on a 650 mm diameter Cu wheel in an air atmosphere (at 30 m/s wheel speed, casting at 1250–1260 °C). The primary alloys were produced from pure chemical elements (Fe(3 N), Co(3 N), Cu(4 N)) and FeB_18_(2.5 N) alloy using induction furnace SecoWarwick VIM-LAB 50–60. For magnetic measurements, the amorphous ribbon was wound into a toroidal core with an inner diameter of about 20 mm and an outer diameter of about 30 mm. Then, the long-term annealing of the toroidal cores was isothermally performed over up to 300 h both in a vacuum furnace (5 × 10^−3^ mbar) and in the air at two different temperatures: 210 and 260 °C. The conventional vacuum annealing process took 20 min at 310 °C. Structural properties of annealed alloys were studied by the X-ray diffraction (XRD) method. XRD measurements were performed at room temperature using a Rigaku MiniFlex 600 diffractometer equipped with CuK_α_ radiation (λ = 1.5406 Å), K_β_ Ni filter and the D/teX Ultra high-speed silicon strip detector.

Dynamic mechanical analysis (DMA) was utilized to verify the presence of primary, secondary relaxation and crystallization, storage modulus, and loss modulus variation versus temperature using Netzsch DMA242E Artemis. Deformation mode, frequency and heating rate were tensile, 1–10 Hz and 2 K/min, respectively.

The differential scanning calorimetry (DSC) was conducted using Netzsch DSC 214 Polyma to measure the crystallization peaks at a 2 K/min heating rate. The isothermal kinetics study of primary crystallization (α-Fe(Co)) has been performed by isothermal measurements at four different temperatures: 330, 340, 350 and 360 °C, where the crystallization process is evident on DSC signal. For temperatures 210 and 260 °C, this process is extremely extended in time, and process is explained only at higher temperatures.

To determine the coercivity and the magnetic saturation hysteresis loops were obtained up to 5000 A/m (defined here as Bs) at 50 Hz, and the Remacomp C-1200 magnetic measurement system (MAGNET-PHYSIK Dr. Steingroever GmbH) was used. Additionally, this system was used to find the maximum magnetic permeability value (µ_max_). The values of core power losses (P_10/50_) for all annealed samples were measured with a magnetic induction B = 1 T and frequency f = 50 Hz. Therefore, for samples annealed at optimum conditions, the core power losses (Ps) were measured under the frequency range of 50 Hz–400 kHz and magnetic induction range of 0.1–0.5 T.

## Results and discussion

**Figure 1:**
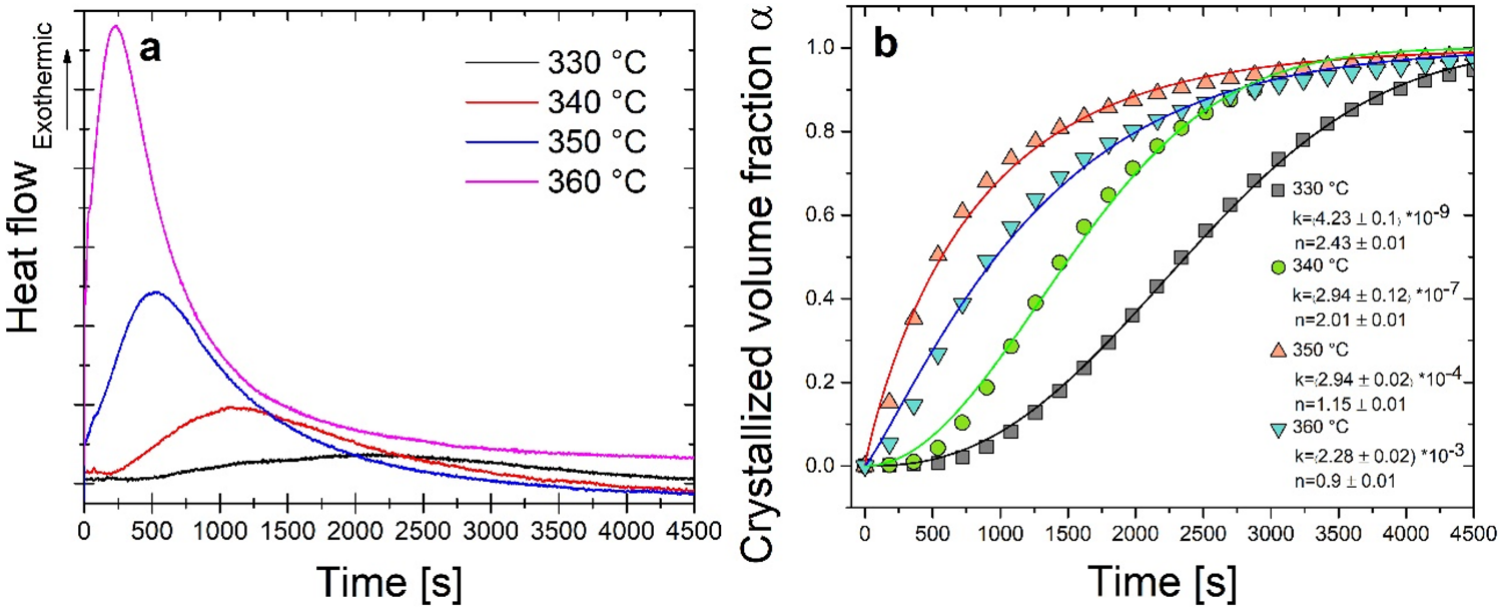
Isothermal kinetics results: (**a**) heat flows; (**b**) Avrami plot with calculated kinetic parameters n and k.

The isothermal crystallization DSC signals of the kinetics of amorphous ribbon at four different annealing temperatures are shown in Fig. 1a. The single exothermic peaks follow a particular incubation period that decreases with the increase in the annealing temperature from 330 to 360 °C. It is usually explained by the higher mobility of atoms at a higher temperature that contributes to a critical fluctuation in concentration to order atoms in the long-range scale for a large scale crystallization process^17^. As a result, the crystallization volume fraction α is directly proportional to the fractional area of the heat flow peak. Based on this calculation, the progression of the crystalline volume fraction versus the annealing time has been presented in Fig. 1b. The curves show the typical sigmoidal shape with their interpretation within the Kolmogorov-Johnson–Mehl–Avrami-Evans (KJMAE) model by two parameters: k—crystallization rate constant and n—Avrami exponent (average index describing the mechanism of crystallization). Both parameters were calculated using the equation^18^:

1 $$\alpha \left(t\right)=1-exp\left[-k{\left(t\right)}^{n}\right],$$

where: α—degree of crystallization, k—crystallization rate constant, n—Avrami exponent, t—time.

This equation can be rewritten as:

2 $$\mathrm{ln}\left(-\mathrm{ln}\left[1 -\alpha \left(t\right)\right]\right)=\mathrm{ln}\left(k\right)+n\mathrm{ln}\left(t\right).$$

A decrease of the Avrami exponent n value from 2.43 to 0.9 and an increase in the k value from 4.23 × 10^−9^ to 2.28 × 10^−3^ s^−1^ with an increase in isothermal annealing temperature were observed. Similar results have already been observed by V.I. Tkatch et al. for the α-Fe phase crystallization in Fe_85_B_15_ amorphous alloy^19^. The value of 2.5 > n > 1.5 at lower temperatures (330 and 340 °C) indicates a growth of crystallites with a decreasing nucleation rate. For temperatures of 350 and 360 °C, the value of n < 1.5 means only diffusion-controlled pre-existing nuclei growth^20^. It may be related to the too intensive growth of crystallites and the rapid blocking of the possibility of forming new nucleation place from the amorphous matrix. The k value is related to the rate of crystallization. Therefore its higher value at higher temperatures means a fast process of crystallization caused by the introduction of a higher energy into the system^18^. Because nucleation and growth change over time, the local Avrami exponent was calculated according to the formula^21^:

3 $$\mathrm{n}\left(\alpha \right)=\frac{\partial \mathrm{ln}(-\mathrm{ln}[1 -\alpha ])}{\partial \mathrm{ln}(t)}.$$

**Figure 2:**
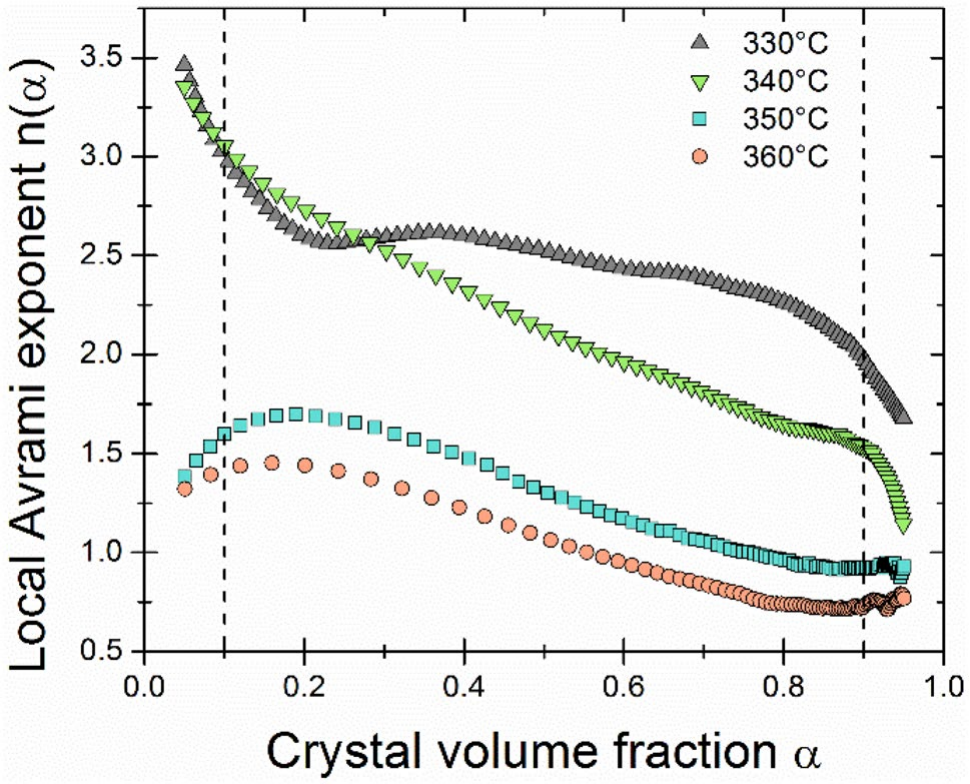
Local Avrami exponents in the function of the crystallized fraction.

The local Avrami exponent n depending on the crystallized fraction is shown in Fig. 2. The values of the local Avrami exponent coincide with the Avrami exponent calculated earlier. In the analyzed range (0.1–0.9 α) for temperature 350 and 360 °C, an initial increase (for α < 0.2) of the n can be noticed, followed by a slight decrease of this value along with further crystallization process. It means that at the beginning of the crystallization process, additional nuclei could be formed in the material, and then only the existing ones grow. For the temperatures of 330 and 340 °C, a high value of n > 3 can be noticed in the initial stage of crystallization, which is related to crystallite growth with an increasing nucleation rate. In the isothermally annealed material at 340 °C, there was an almost linear decrease in the n value with the crystallization progress due to the slower appearance of new nuclei from the amorphous phase. However, for the ribbon annealed at 330 °C, the n value stabilized at 2.5, which means growth with a constant nucleation rate. For both temperatures, a sudden drop in the value of n was observed at the final stage of crystallization. T. Paul et al. observed a similar behaviour of changes in the n exponent with an increase in the crystalline fraction for the primary isochronal crystallization of Fe_48_Cr_15_Mo_14_Y_2_C_15_B_6_ amorphous alloys^20^.

**Figure 3:**
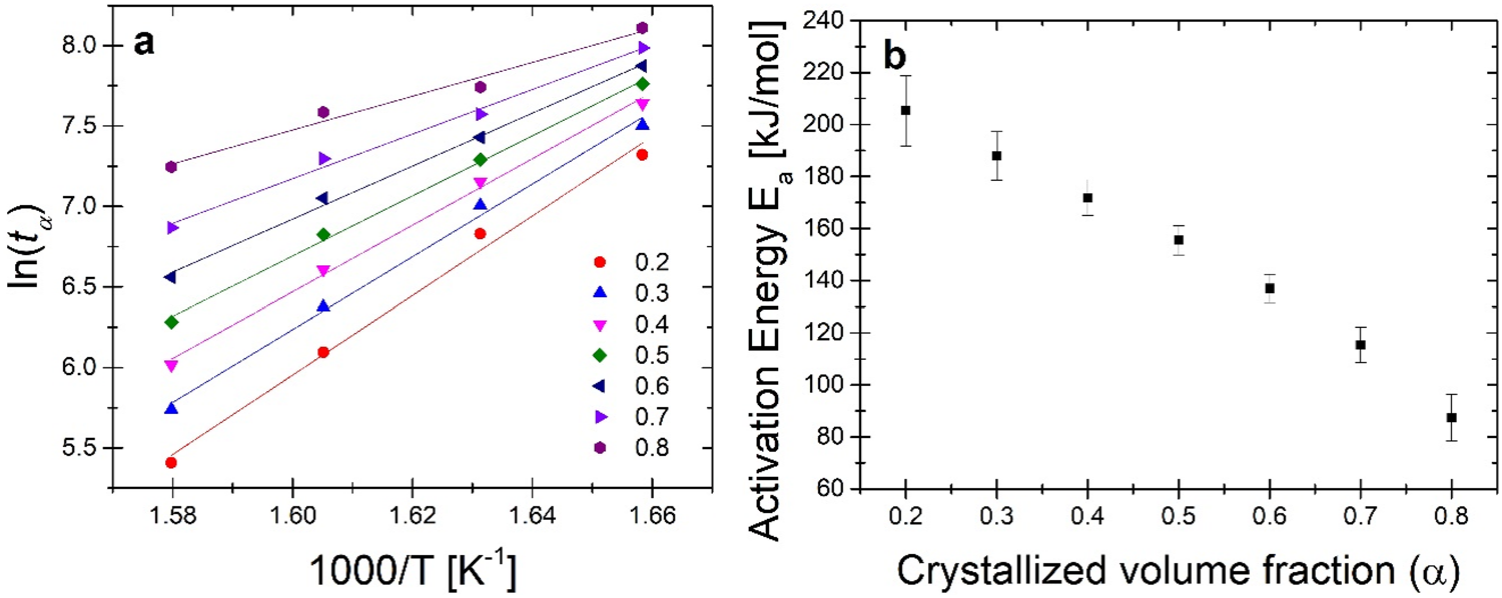
(**a**) Isothermal kinetics results: Arrhenius plots for activation energy determination; (**b**) local activation energy from crystallized volume fraction dependence.

To better understand the α-Fe(Co) phase crystallization process, the local crystallization energy (E_a_(α)) was calculated using the Arrhenius equation^22^.

4 $$\mathrm{t}\left(\alpha \right)={t}_{o}\mathrm{exp}\left[\frac{{E}_{a}\left(\alpha \right)}{RT}\right],$$

where t(α) is the isothermal annealing time at the crystalline volume fraction $\alpha$, t_o_ is a time constant, R is the gas constant, and T is the isothermal annealing temperature. *E*_a_(α) were obtained from the slope of the linear fitting plots of ln[t(α)] versus 1000/T for 0.2–0.8 α presented in Fig. 3a. Figure 3b shows the activation energy E_a_ as a function of the crystallized volume fraction α. An almost linear decrease in the E_a_ value from 205.3 to 87.5 kJ mol^−1^ can be seen with the progress of crystallization. This behaviour should be explained by reducing the energy barrier needed for the crystallization process to take place along with the progress of this process. Similar relationships were observed for Fe_75_Cr_5_P_9_B_4_C_7_ amorphous ribbons with E_a_ changing from 412 to 383 kJ mol^−1^^22^. It is worth noting that the material tested is characterized by over 2 times lower E_a_ and a much greater change in its value during the crystallization process.

**Figure 4:**
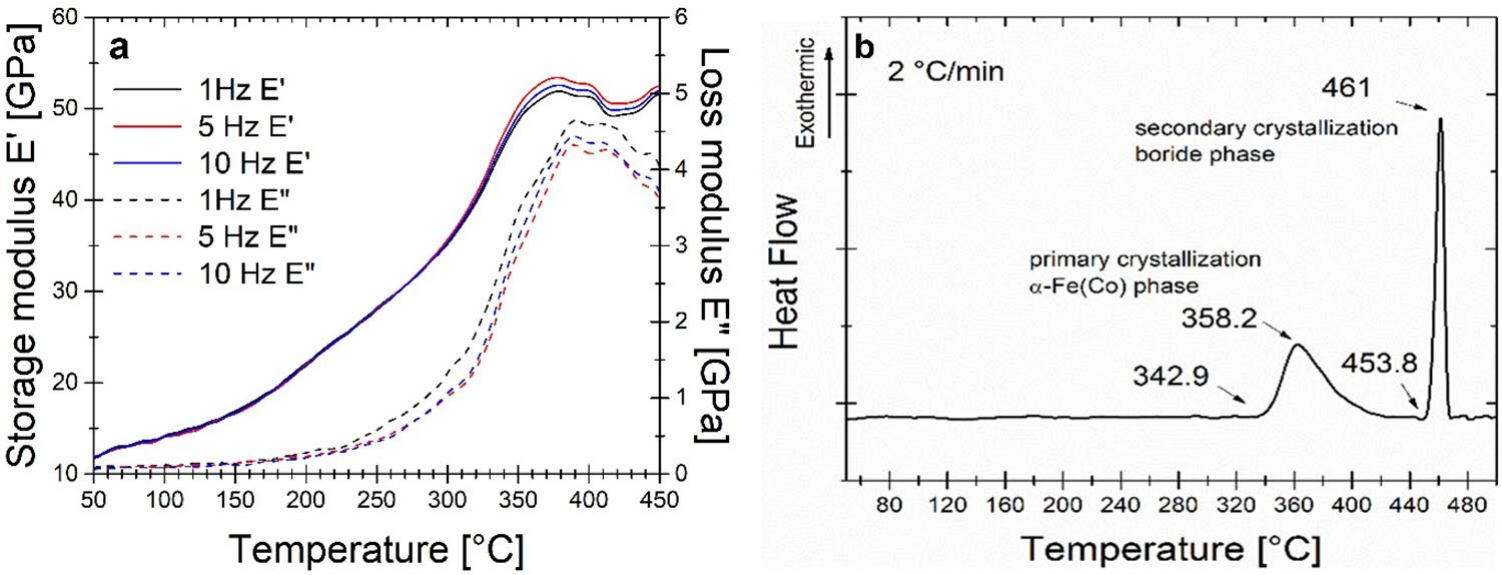
(**a**) DMA results of storage modulus E’ and loss modulus E” at the heating rate 2 °C/min; (**b**) DSC signal at the heating rate of 2 °C/min.

Dynamic mechanical analysis was used to verify the presence of relaxation processes in the glassy state during annealing. There is a well-known problem of finding the glass transition temperature (Tg) in Fe-based amorphous ribbons in the heat flow signal. It is usually unidentified because of the very weak effect, and materials crystallize almost immediately after the glass transition is passed^23^. There is only very limited number of ferromagnetic glassy alloys with a wide supercooled liquid region^24^. Figure 4a shows the temperature dependence of the storage modulus E’ and loss modulus E”. For temperatures below 250 °C, the ribbon is slightly stress relieved with increasing value of E’ with the minor change of E”. However, this relieving is almost independent of frequency change and does not possess the typical behaviour of secondary relaxation. At a temperature from 250 to 380 °C the substantial increase of both E’ and E” takes place what is usually explained by the large scale atoms displacement (named as α-relaxation) with subsequent crystallization process of α-Fe(Co) phase, that is proved by DSC results presented in Fig. 4b. The onset temperature of α-Fe(Co) phase crystallization exists at 343 °C. Finally, brittleness increases during the crystallization process until the sample breaks. Amorphous materials are generally isotropic and homogenous on a macroscale. However, going into microscale, many heterogeneities occur that is usually detected by diversity in density. Such a non-equilibrium state is compositionally short-range ordered while annealing by Cu-clustering^25,26^ before the crystallization process leading to an increase in the local concentration of Fe in the vicinity of these clusters and the formation of bcc-Fe crystals. Previously presented study for Si-content^26^ alloys showed that storage modulus E’ in the amorphous state before α-relaxation and crystallization process is much higher E’ = 40–50 GPa in the wide range of temperature than for Si-free alloy presented here and increase with Si content up to 120 GPa^26^. Co for Fe substitution substantially changes the mechanical properties, including hardness and elastic modulus, which also decreases the storage modulus E’ of the studied material.

**Figure 5:**
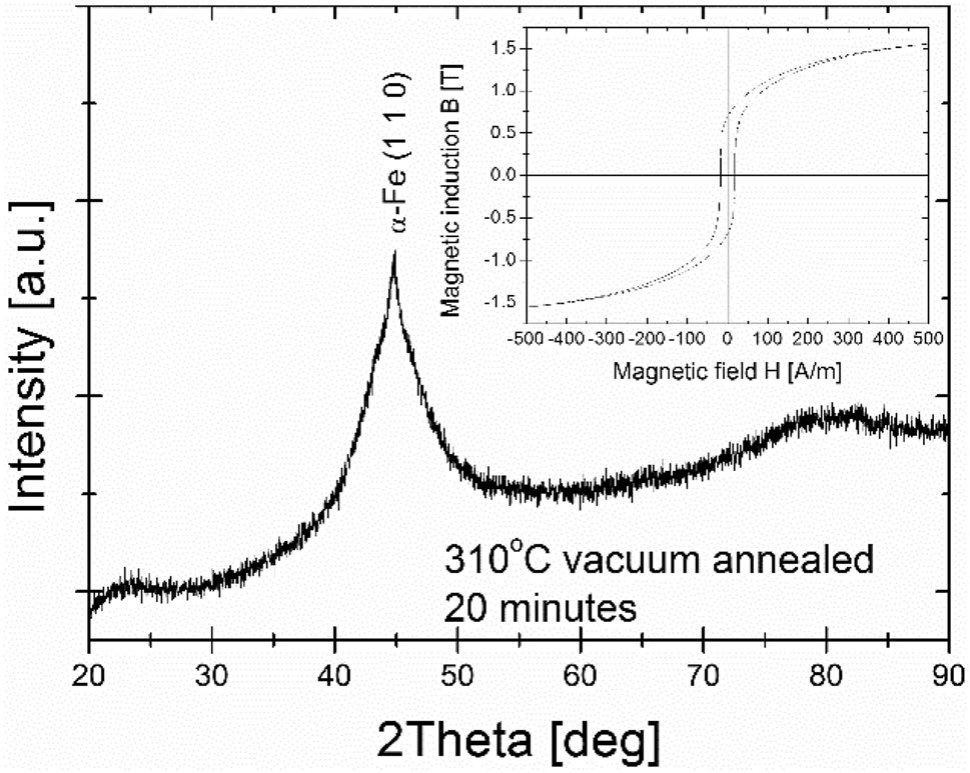
X-ray diffraction pattern and B-H hysteresis loop as the figure inset of conventionally vacuum annealed sample at 310 °C for 20 min.

**Figure 6:**
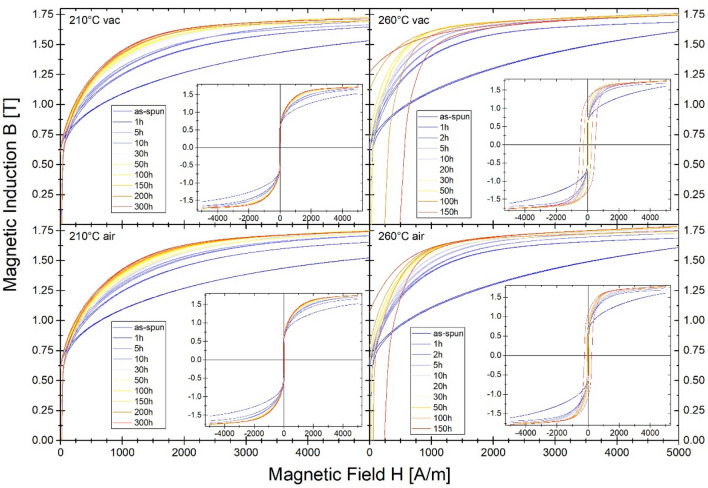
Evolution of hysteresis loops B(H) for vacuum and air annealed materials at 210 °C and 260 °C in different processing time.

**Figure 7:**
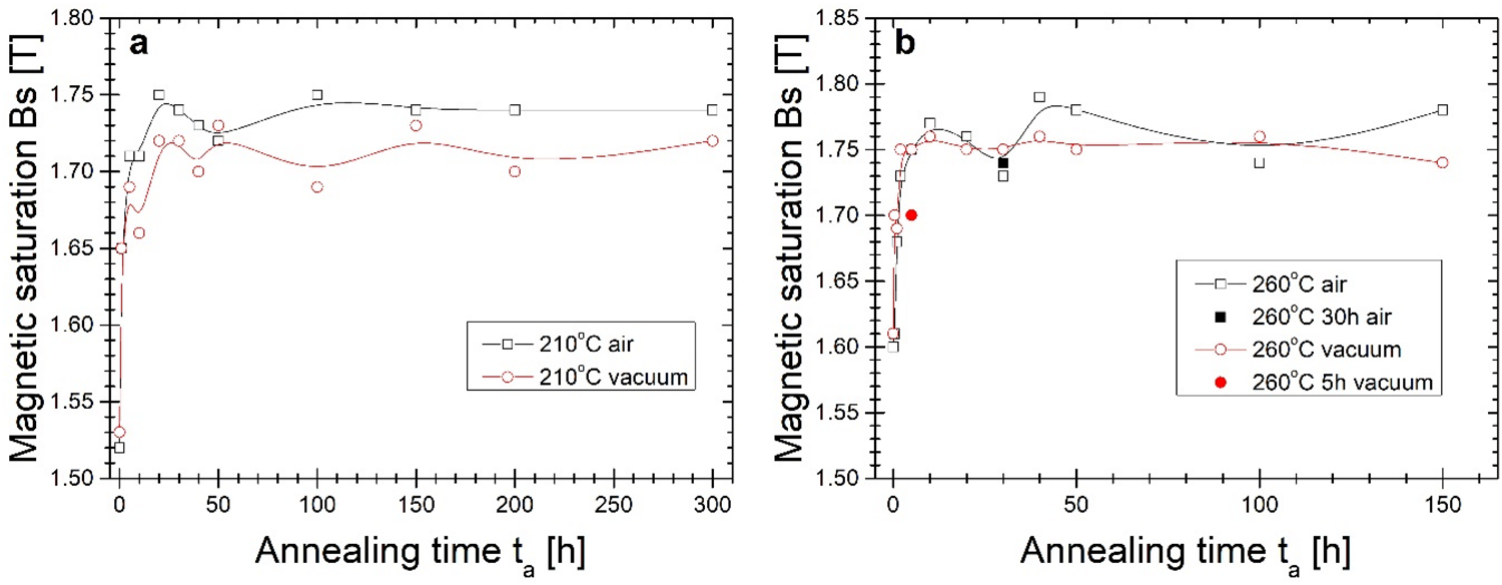
Magnetic saturation on the annealing time of the isothermal process at (**a**) 210 °C and (**b**) 260 °C.

**Figure 8:**
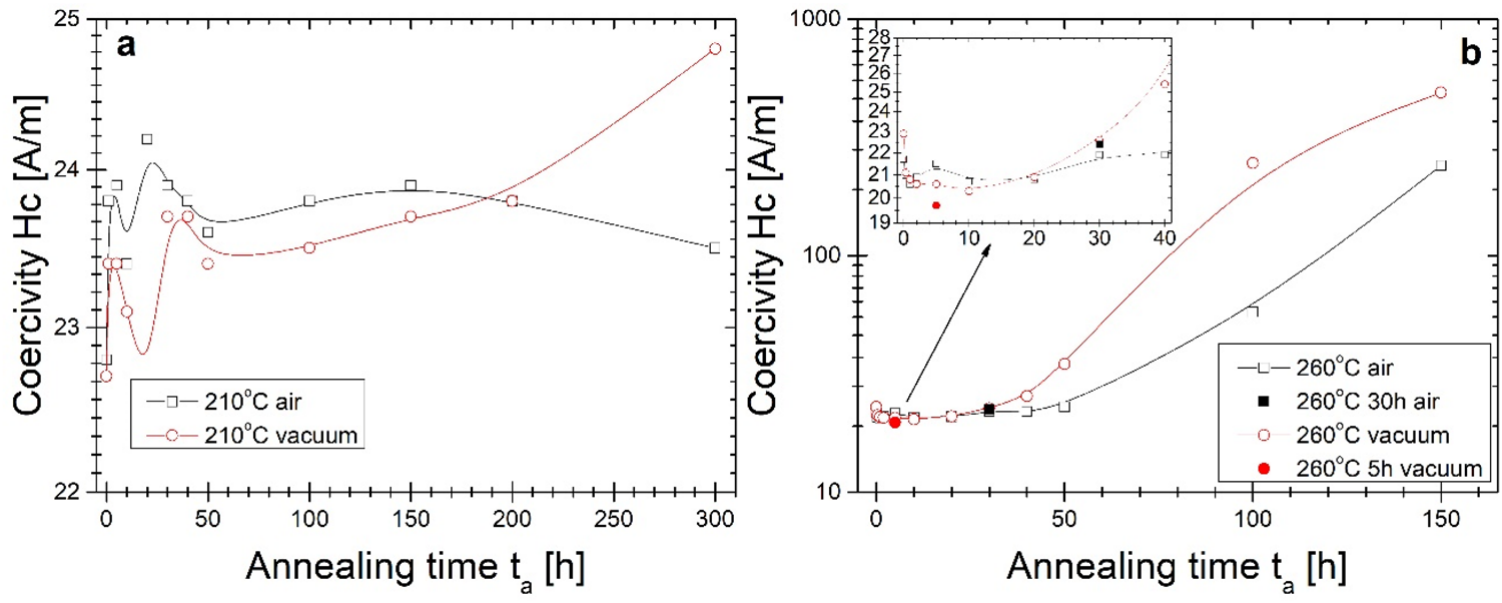
Coercivity on the annealing time of the isothermal process at (**a**) 210 °C and (**b**) 260 °C.

**Figure 9:**
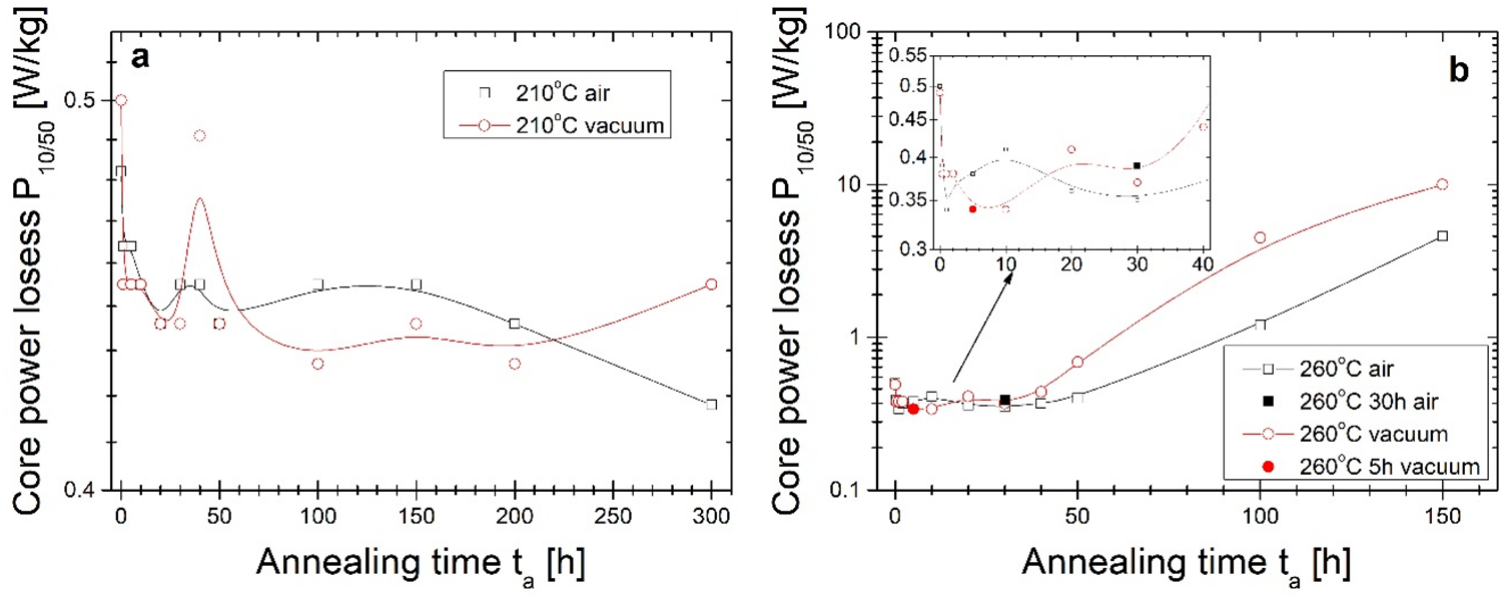
Core power losses P_10/50_ on annealing time of isothermal annealing process at (**a**) 210 °C and (**b**) 260 °C.

The optimal, from a magnetic point of view (the least lossy magnetic state with minimum value of P_10/50_), the 20 min isothermal annealing process has been found at 310 °C. The crystal structure study performed via the XRD method proved the presence of an early-stage crystallization process (Fig. 5). In this work, the B(H) hysteresis loops (Fig. 6) and resulting therefrom saturation induction Bs (Fig. 7), coercivity Hc (Fig. 8) and core power losses P_10/50_ (Fig. 9) of Fe_80.3_Co_5_Cu_0.7_B_14_ alloy long-term up to 300 h vacuum- and air-annealed at two different temperatures 210 and 260 °C are presented. It can be seen that the as-quenched alloy is on the initial Bs level of 1.5–1.6 T. Bs values for both temperatures and air- and vacuum-annealing substantially increase with annealing time (t_a_) up to first Bs maximum state, then fluctuate. The air-annealing process at both temperatures allows to obtain higher Bs values. However, because of the surface oxidation process, the annealing process seems to be slightly delayed compared to vacuum annealing. First Bs = 1.77 T maximum state for air-annealed alloy at 260 °C exists after t_a_ = 5 h, while the second Bs = 1.79 T after 40 h. For vacuum-annealed alloy, the Bs(t_a_) dependence is much more flat after 2 h of annealing and fluctuates later around 1.75 T. For alloy annealed at 210 °C, Bs increases during the first 20 h of air- (up to 1.75 T) and vacuum-process (up to 1.72 T). After 20 h of annealing up to 300 h, the Bs value stabilizes around 1.74 T in an air and around 1.71 T in a vacuum.

Going into more profound insight into Hc(t_a_) and P_10/50_(t_a_) (Figs. 8, 9) dependences it can be seen the relatively wide t_a_ range (2–30 h) plateau of low Hc and P_10/50_ values for air- and vacuum-annealed alloy at 260 °C with a minimum value of P_10/50_ = 0.34 W/kg after 5 h annealing in vacuum, and after 30 h annealing in air. Coercivity value fluctuates in the plateau region on the level 20–21 A/m. For more extended heat treatment, the substantial increase of Hc and P_10/50_ values take place. However, for air-annealed alloy, this increase is delayed in time. For annealing at 210 °C, Hc(t_a_) and P_10/50_(t_a_) dependences show the relatively fast stabilization of both values on coercivity levels at about 23–24 A/m and core power losses at 0.43–0.45 W/kg. Such values are higher than those obtained for alloy annealed at 260 °C. For minimum values of core power losses obtained at 260 °C after 5 h in a vacuum and 30 h in air, the heat treatment process has been repeated as one-step isothermal annealing for 5 h in a vacuum and 30 h in the air. Results are marked on pictures by full symbols. Hc and P_10/50_ are on the same level of value, and only Bs for the vacuum-annealed alloy is 0.05 T lower than for the multi-step annealing process.

**Figure 10:**
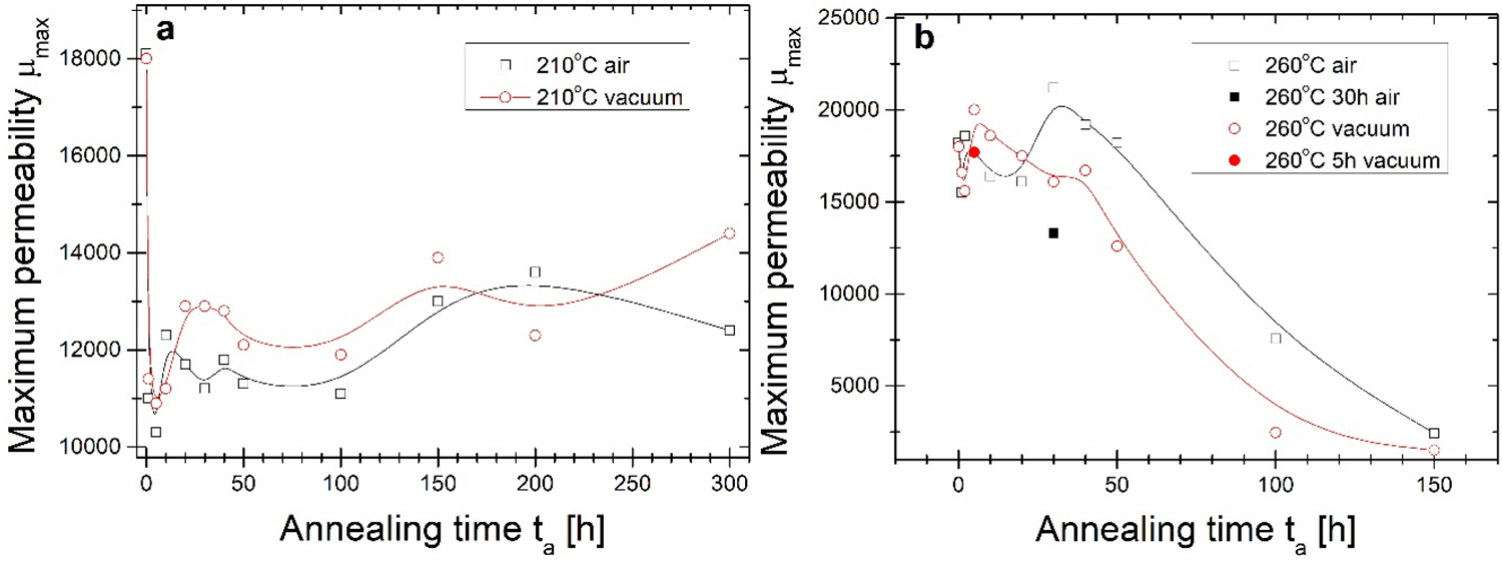
Maximum magnetic permeability on the annealing time of the isothermal process at (**a**) 210 °C and (**b**) 260 °C.

**Figure 11:**
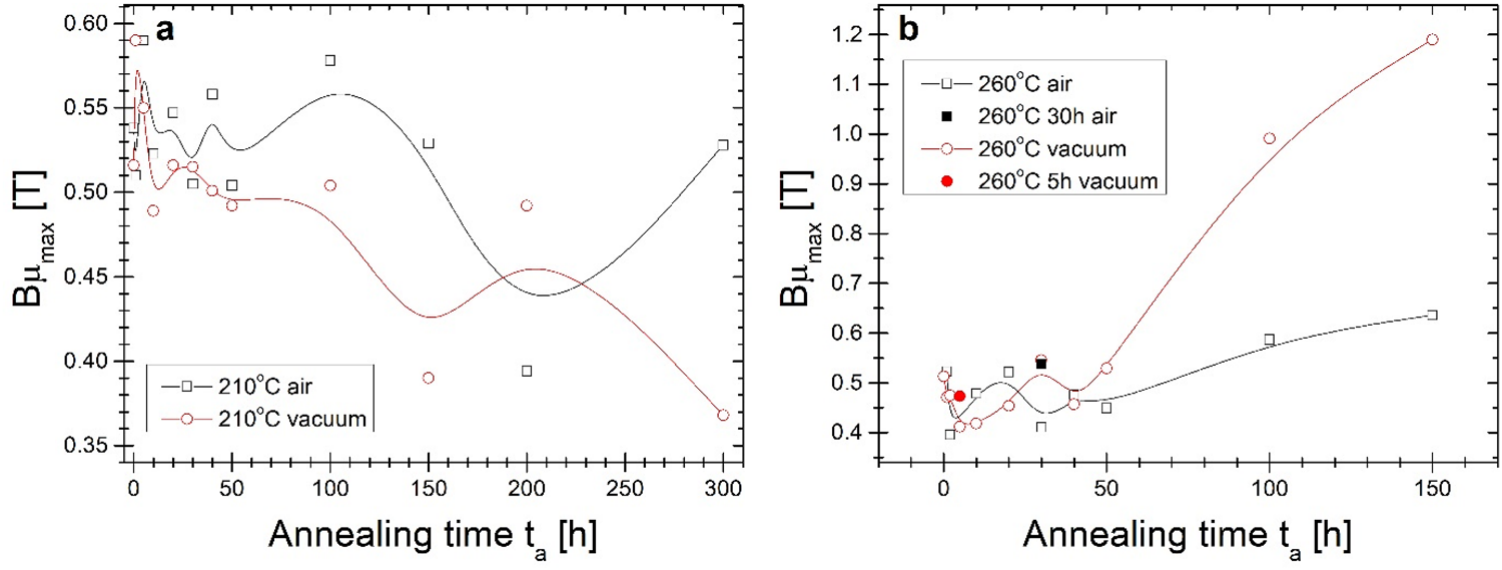
Bµ_max_ on the annealing time of the isothermal process at (**a**) 210 °C and (**b**) 260 °C.

The comprehensive studies of long-term annealing influence on maximum permeability findings correlate with magnetic induction at frequency 50 Hz. Figure 10 shows maximum magnetic permeability µ_max_ from annealing time, while Fig. 11 the magnetic induction B at µ_max_ from annealing time dependence. As it is clearly shown in Fig. 10 the initial value of µ_max_ for cores before annealing is between 17,000–18,000. For both annealing temperatures, µ_max_ value firstly decreases what suggests the initial quench-in stress relief. However, this decrease is two times greater for T_a_ = 210 °C. After the initial decrease of µ_max_ related with stress relief for both alloys, air- and vacuum-annealed µ_max_ successively increases during 300 h of annealing up to µ_max_ close to 14,000. Some values’ fluctuation can also be observed. For T_a_ = 260 °C after initial stress relief in the first step of annealing, there is a substantial increase for vacuum-annealed sample after 5 h up to 20,000, then µ_max_ slowly decreases with t_a_ during the next 25 h of annealing followed by a significant drop in µ_max_ dependence with annealing time up to µ_max_ = 2000 for 150 h of annealing. For the air-annealing process, the µ_max_(t_a_) dependence there is local maximum µ_max_ = 18,000 after 5 h of annealing and a global maximum of µ_max_ = 22,000 after 40 h of annealing. For t_a_ from 50 to 150 h, a significant drop in µ_max_ is seen, similarly to vacuum-annealed alloy. Soft magnetic properties strongly deteriorate in this annealing period. The one-step annealing process performed for 5 and 30 h in vacuum and air respectively proves that surface oxidation limits the formation of soft magnetic properties in the one-step air-annealing process.

In Fig. 11 the magnetic induction value at µ_max_ (Bµ_max_) from annealing time dependences are presented. Both annealing processes performed at lower temperature maximum magnetic permeability measured at 50 Hz are measured for magnetic induction range of 0.4–0.6 T. It is positively assessed that maximum of magnetic permeability shifts to a lower B value. For Ta = 260 °C for the same annealing period up to 50 h where high µ_max_ exists in Fig. 10 the magnetic induction varies from 0.4 to 0.55 T. However, the minimum value exists for annealing time with the highest µ_max_ value. For annealing time 100 and 150 h, the magnetic induction at the maximum permeability value is closer to the saturation induction up to 1.2 T for vacuum-annealed for 150 h.

**Figure 12:**
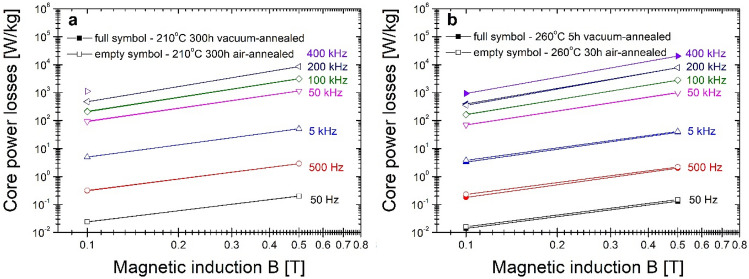
Core power losses in the function of magnetic induction for annealed metallic ribbons at (**a**) 210 °C and (**b**) 260 °C.

All magnetic parameters mentioned above have been measured for frequency f = 50 Hz. From the application point of view, especially for high-Bs alloys studied in this work, the crucial is knowledge of magnetic parameters in the frequency range up to middle and high frequencies. Thus additional measurements have been performed at two selected states of annealing: final state after 300 h of annealing at 210 °C (presented in Fig. 12a) and in the least lossy state at 260 °C determined from Fig. 9 (e.g. after 5 h of vacuum annealing and 30 h of air-annealing) (presented in Fig. 12b). From the figures inspection, it can be noticed that there are almost no differences between the air- and vacuum-annealing. Because of log–log scales, the direct visual comparison between both temperatures is complex. In Table 1 the core power losses of the vacuum-annealed alloy at 210 and 260 °C studied in this work are compared with annealed alloy at 310 °C for 20 min. This comparison shows that the minor lossy state comes from 20 min of annealing at 310 °C. The optimal annealing at 260 °C gives almost two times higher lossy material in the frequency range from 100 to 400 kHz, and also in lower frequency range lower temperature of annealing gives higher lossy material. For Ta = 210 °C, the Ps values are even higher. However, the difference between Ps values for 210 and 260 °C is not very substantial.

**Table 1:** Comparison of core power losses measured at B = 0.1 T in the frequency range from 50 Hz to 400 kHz.

Frequency	Ps [W/kg] 210 °C/300 h	Ps [W/kg] 260 °C/5 h	Ps [W/kg] 310 °C/20 min
50 Hz	0.024	0.014	0.011
500 Hz	0.32	0.18	0.13
5 kHz	5	3.3	2.1
50 kHz	92	70	38
100 kHz	207	168	82
200 kHz	477	394	200
400 kHz	1124	941	534

**Figure 13:**
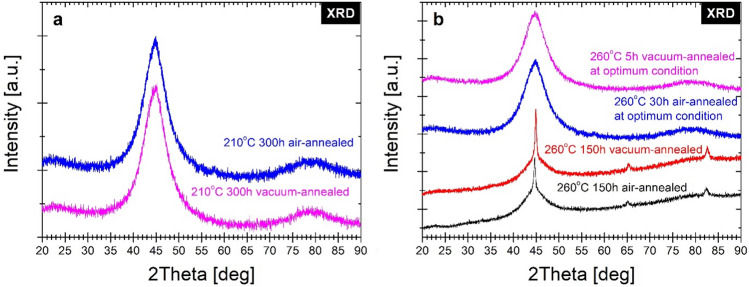
X-ray diffraction patterns of annealed ribbons at (**a**) 210 °C and (**b**) 260 °C.

To verify the crystal structure state for both annealing temperatures the XRD measurements have been performed at final states of multi-step annealing (after 300 h at 210 °C, after 150 h at 260 °C) and also for optimally one-step annealed (after 5 h of vacuum-annealing at 260 °C, after 30 h of air-annealing at 260 °C) alloy. From the inspection of XRD patterns gathered in Fig. 13a the final state of the crystal structure annealed at 210 °C (after 300 h) exists still in the amorphous state, and only diffused amorphous diffraction halos are seen. Similarly, for optimally annealed at 260 °C alloys (Fig. 13b), the diffraction patterns prove the amorphousness of the materials within the method accuracy. The XRD patterns (Fig. 13b) of 150 h air- and vacuum-annealed alloys at 260 °C prove the presence of well-crystallized the α-Fe(Co) phase with a small contribution of the amorphous matrix as a residual diffused diffraction halos.

From the correlation of magnetic properties and crystal structure changes during long-term low-temperature annealing, it has to be noticed that the first process of isothermal annealing at 210 and 260 °C is related with quenched-in stress relief that is related to a substantial initial increase of Bs with simultaneous decrease of P_10/50_ and µ_max_. From the magnetic point of view, eliminating internal quenched-in stresses can improve the mobility of the Bloch wall of the magnetic domain^27^, and the magnetic anisotropy starts to fluctuate during the change of the topological short-range order^28^. From the point of view of the crystal structure, ageing in metastable quenched metallic glasses induces a lower enthalpy, a smaller volume, a more stable glassy state, and changes the topological short-range order, which is characteristic for the glass structure^29,30^. During 300 h isothermal annealing process at 210 °C, the slight trend of magnetic parameters changes is seen as a decrease in core power losses, increased maximum permeability, and decreased magnetic induction for which its maximum occurs. XRD studies proved the presence of only a glassy state after 300 h of annealing. However, slow changes in magnetic properties mentioned above suggest changes in compositional short-range order. Some of the previous studies have shown that the relaxation process of FeNiSiB systems can be divided into two stages: the first—metalloid atoms movement, the second—diffusion of the constituent atoms^31^. According to the Fe–Co–Cu-B system study with relatively high B = 15% content and the highest mobility of its atom in the system, its migration plays a crucial role in the first step of glass relaxation, density equalization, volume minimization. Then the Cu clustering process was slightly reduced by less favourable Cu and Co mixing enthalpy (6.5 kJ/mol) than for Cu and Fe (13 kJ/mol)^32^. Additionally, the Co atom has a lower radius than the Fe atom and is more mobile during atomic rearrangement. Going into an interpretation of magnetic properties evolution during long-term annealing at 260 °C it is seen that after the first step quenched-in stress relief during the next 30 h of annealing, the glass relaxation in an amorphous state takes place. From 40 to 50 h of annealing, the substantial change of magnetic parameters is seen in Hc(t_a_), P_10/50_(t_a_), µ_max_(t_a_) dependences. The soft magnetic character deteriorates. However, the Bs value remains on the level 1.75–1.77 T. According to the random anisotropy model (RAM) proposed by Herzer^33^, the Hc is proportional to the fourth power of magneto-crystalline anisotropy and six power of mean grain size. Based on information from XRD measurements, in the final state, after 150 h of annealing, only α-Fe(Co) exists with the dominant content of the amorphous matrix. The compositional short-range ordering occurs during the annealing process, and atomic clusters are formed within the amorphous matrix. The coupling of these clusters leads to anisotropy. However, it should be noticed that air-annealing by the presence of oxidized coating formed during annealing leads to higher Bs values up to 1.79 T, and the surface oxidized alloy is more resistant to soft magnetic properties deterioration. Magnetic properties and crystal structure evolution investigated here during 150 h of annealing correspond approximately to the ones presented during isochronal 20 min of annealing at 310 °C. However, by directly comparing core power losses from frequency dependences, the long-term annealing is energetically insufficient to bring the glassy state system into the same low level of core power losses. To understand all the correlations between the structural details and magnetic behaviour a deeper insight into a local short-range atomic-scale order via in-situ temperature transmission electron microscopy and magnetic domain observations should be taken.

## Conclusions

Isothermal kinetics of crystallization process, crystal structure and magnetic properties (Bs, Hc, P_10/50_, µ_max_ and Bµ_max_) evolution during long-term low-temperature annealing were investigated using DSC, DMA, XRD and magnetic measurements. As a result, the following conclusions are drawn:

- (1) The parameters of crystallization α-Fe(Co) phase kinetics for isothermal annealing have been determined. With a decrease in isothermal annealing temperature, the growth of crystallites occurs with a decreasing nucleation rate. The calculated activation energy of this crystallization process decreases almost linearly with crystallized volume fraction from 205.3 to 87.5 kJ/mol.
- (2) In temperature range from 250 to 380 °C a substantial increase of both E’ and E” takes place that is usually described by the large scale atoms displacement (named as α-relaxation) with subsequent crystallization process of α-Fe(Co) phase, DSC results prove that. Below 250 °C, the material is stress-relieved with increasing value of E’ and the minor change of E”.
- (3) Magnetic properties and crystal structure evolution investigated here during 150 h of annealing at 260 °C correspond approximately to the ones presented during isochronal 20 min of annealing at 310 °C. Magnetic properties Bs = 1.75–1.79 T, Hc < 20 A/m, and P_10/50_ are similar to those for optimum classical annealing.
- (4) During 300 h isothermal annealing process at 210 °C, the slight trend of magnetic parameters' changes is seen as a decrease of core power losses, increased maximum permeability, and decreased magnetic induction for which its maximum occurs. The long-term annealing is energetically insufficient to bring the glassy state system into the same low level of core power losses efficiency as for 20 min annealing at 310 °C.

## Data Availability

The datasets used and/or analysed during the current study available from the corresponding author on reasonable request.
